# Editorial: Safeguarding youth from agricultural injury and illness: international experiences

**DOI:** 10.3389/fpubh.2023.1270578

**Published:** 2023-08-18

**Authors:** Barbara C. Lee, Florence A. Becot, Casper Bendixsen, Christopher Benny, Peter Lundqvist, Andrea Swenson, Bryan Weichelt, Richard C. Franklin

**Affiliations:** ^1^National Children's Center for Rural and Agricultural Health and Safety, Marshfield, WI, United States; ^2^National Farm Medicine Center, Marshfield, WI, United States; ^3^Medical College of Wisconsin, Wausau, WI, United States; ^4^Department of People and Society, Swedish University of Agriculture Sciences, Uppsala, Sweden; ^5^College of Public Health, Medical and Veterinary Sciences, James Cook University, Townsville, QLD, Australia

**Keywords:** children, youth, agriculture, farm, work, safety, injury, policy

Worldwide, agriculture is among the most dangerous industries and one of the few that involves children (< 18 years-of-age) in the worksite as laborers or bystanders (1). Children are exposed to an array of agriculture-related hazards whether working or merely being present in the farm environment. From a public health and child advocacy perspective, safeguarding these young people from preventable disease and injury is important for many reasons. The negative impacts of a childhood agricultural disease or injury range from permanent disabilities, death, family disruptions, and economic hardships including the potential loss of a sustainable family farm enterprise (2). At the same time, growing up in an agricultural setting can lead to independent, hardworking, successful adults, who gain a range of benefits, including skill development, family time together, improved immune response, and other protective health factors (3, 4).

Interest in agricultural occupational safety gained traction in the 1950s as awareness of the preponderance and preventability of fatal and non-fatal farm injuries grew, combined with increased industrialization and mechanization in agriculture, and the increasing science around occupational health and safety. However, despite the family farm model being the most prevalent structure in agriculture worldwide, it was not until the early 1990s that a growing interest in child safety on farms culminated in the first major symposium on childhood agricultural injury prevention. Convened in the United States, this symposium brought together a range of stakeholders, including researchers, educators, and advocates with different perspectives, and was key to launching a network of stakeholders around the goal of ensuring the safety of children on farms (5).

In 1997, the National Children's Center for Agricultural Health and Safety (NCCRAHS) (https://www.marshfieldresearch.org/nccrahs) was established with funding from the US National Institute for Occupational Safety and Health. To our knowledge, this US-based center is the only research and outreach center funded by a national government that is dedicated solely to agricultural health and safety of children. As NCCRAHS celebrates its 25th anniversary, this special *Frontiers in Public Health* issue provides an opportunity to take stock of the childhood agricultural injury prevention scholarship globally and to reflect on opportunities to move forward. Ultimately, our goal with this Research Topic is to strengthen international collaborations to reduce the toll of preventable childhood farm injuries and illness.

In this Research Topic, we provide 28 papers that explore a wide spectrum of issues associated with safeguarding youth from agricultural injury and illness. These include two sets of papers: (1) solicited commentaries providing country-specific descriptions of the current situation of child and adolescent safety on farms and ranches, along with recommendations for the future; and (2) submitted papers, including original research, literature/policy/protocol reviews, brief research reports, and case studies. The editorial team's objectives were to: (1) reach out to international communities to identify organized efforts focused on childhood agricultural safety; (2) encourage individuals or groups to assess and document the status of efforts in their respective countries; (3) publish relevant commentaries, interventions, and research; and (4) synthesize findings and identify opportunities for collaborative strategies to address complex issues. As we embarked on this effort with *Frontiers in Public Health*, we reached out to as many different countries as possible, seeking diverse international perspectives on this topic. Through our collegial networks and internet searches, we made individual contacts with potential authors representing 31 different countries. It was disappointing to hear from colleagues in some countries such as France, Thailand, and Turkey that the topic of children and agricultural safety receives limited or no attention in their country, even when agriculture plays a key role in their economy. Overall, the submissions we received provide descriptive explanations of injury data (or lack thereof), major agents of agriculture-related injury, key concerns, and policy issues from eight countries across five continents.

These research findings, educational program descriptions, brief reports, and commentaries will take you on a journey across a wide range of issues. They include the most current data regarding child farm injuries and fatalities from publicly available news media (Weichelt et al.), youth's (un)willingness to work in the agricultural sector (Girdziute et al.), legal responses to traumatic injuries associated with child endangerment (Benny et al.), specific hazards such as all-terrain vehicles (Godler et al.; Brumby et al.), noting that there is a move to call them quadbikes, as it gives an impression that they can be driven anywhere (6), parents' perspectives of children working with livestock (Klataske et al.), to mental health of farm adolescents and their parents (Rudolphi and Berg). A unique aspect of this Research Topic is the international commentaries from eight countries (Maïga and Traoré; Grigioni; Lundqvist; Franklin; McNamara, Mohammadrezaei, Griffin; Pickett et al.; Shortall; Lee and Salzwedel), plus a report from Nigeria about the need for chemical safety training (Udoh and Gibbs), the challenges of agricultural safety in Africa's nation of Burkina Faso, who have recognized the rights of children but do not have specific laws around children working in agriculture (Maïga and Traoré), and/or the laws are not uniformly enforced, as in Argentina (Grigioni).

While the paucity of data was a reoccurring theme (Weichelt et al.; Grigioni; Franklin; Peden et al.), researchers from Australia (Adams et al.) have been working toward having a set of validated questions to help identify risks and effective prevention strategies on farms, while US researchers explore the use of existing surveillance systems to extract relevant data (VanWormer et al.). There are papers exploring new but also persistent areas such as youth's (un)willingness to work in the agricultural sector with authors from Lithuania, Finland, and Germany (Girdziute et al.), discussing the need to better describe positive opportunities and ensure training programs that link agriculture with nature and a love of animals (Klataske et al.), and the negative impact that peers sometimes have on farm injury risk perception of adolescents (Mohammadrezaei et al.). Another study revealed the invisibility of the lived realities of raising children on farms and lack of programming to help farm parents navigate the practical aspects of childcare despite childcare being a key farm safety strategy (Becot et al.). Two papers are about existing guidelines, Agricultural Youth Work Guidelines and Gear Up for Ag Health and Safety^TM^ program, which are intended to provide options to safely incorporate children in farm work while they gain valuable work experiences (Brumby et al.; Swenson et al.). In contrast, youth's involvement in unsafe and extended hours of work is associated with failure of labor laws to protect them (Iannacci-Manasia). Another emerging issue is the increased number of public health emergencies, often associated with climate change, that impact youth in agriculture, and this concern is explored in a paper from Puerto Rico (Pagán-Santana et al.).

Benny et al. in their paper, describe the process for identifying legal responses to child endangerment on farms, comparable to non-farm cases, noting the purpose is not to induce punishment but to influence a culture of safety via a restorative justice process. This links to the work from Ireland, which demonstrates that children on farms are risk factors for adult workers (McNamara, Mohammadrezaei, Dillon, et al.). A survey in the US revealed there is a need to improve the dissemination of child farm safety resources (Salzwedel et al.), including the safety guidelines for youth agricultural work (Swenson et al.). It was also interesting to see new technologies being incorporated into farm safety education via augmented reality (Namkoong et al.) and the use of social media platforms such as Facebook for rural research participant recruitment (Burke et al.).

Several cross-cutting issues were noted in these papers. The lack of reliable, timely agricultural injury and fatality data is a common international theme for both adults and youth. The shortcomings of public policy and regulations seemed to be prevalent across all countries, and this was specifically highlighted in papers from the US (Lee and Salzwedel; Becot et al.; Iannacci-Manasia), Israel (Godler et al.), Ireland (McNamara, Mohammadrezaei, Griffin), Canada (Pickett et al.), and the United Kingdom (Shortall). Finally, the use of common terminology was encouraged. In countries where the discipline of injury prevention has matured, the word “accident” is avoided whenever possible, because the scientific discipline of injury prevention views fatal and non-fatal injuries as predictable and preventable. In the English language, the term “accident” implies a random, unavoidable event or act of God. The concept that injuries are not accidents is widely supported in the literature (Salzwedel et al.). Thus, the editorial team gave instructions to authors to replace the term “accident” with other descriptive words such as injury event, incident, or details such as a crash or suffocation.

Safeguarding youth in agriculture is a complex issue, partly because of the complexity and diversity of farming, lax and inconsistent child labor regulations, international labor trafficking and servitude, economic hardships, limited childcare options, culture and tradition, a disconnect between knowledge of injury risks and actual safety practices, complex public policy frameworks, and changing developmental characteristics as children age. There is still a long way to go to reduce the toll of childhood farm injuries and illness. This *Frontiers* issue, with authors from 15 countries, reveals commonalities across borders and opportunities for collaboration. Perhaps, just as important, this Research Topic highlighted regions of the world where far more attention to children in agriculture is warranted. We hope child safety advocates in these locations can leverage the existing research and initiative to help secure the resources they need to address this topic. Policymakers also have a key role to play. Adequate and consistent funding is needed for effective research, outreach efforts, and community-based initiatives such as off-farm childcare programs. Public policy is also important to support evidence-based interventions such as minimum age for operating farm machinery on public roads or handling pesticides. We view our Research Topic of *Frontiers in Public Health: Safeguarding youth from agricultural injury and illness: international perspectives* as a call for greater international collaboration to ensure the safety of children and youth who live and/or work in agricultural settings across the globe.

## Author contributions

BL: Conceptualization, Funding acquisition, Writing—original draft, Writing—review and editing. FB: Writing—review and editing. CBend: Writing—review and editing. CBenn: Writing—review and editing. PL: Writing—review and editing. AS: Writing—review and editing. BW: Writing—review and editing. RF: Writing—original draft, Writing—review and editing.
